# Supplementary figures and images for: Female sexual agency and frequent extra-pair copulations, but no extra-pair paternity, in Nazca boobies (Sula granti)
Source: PLoS One. 2025 Oct 30;20(10):e0324762. doi: 10.1371/journal.pone.0324762 (PMC12574908; doi:10.1371/journal.pone.0324762)

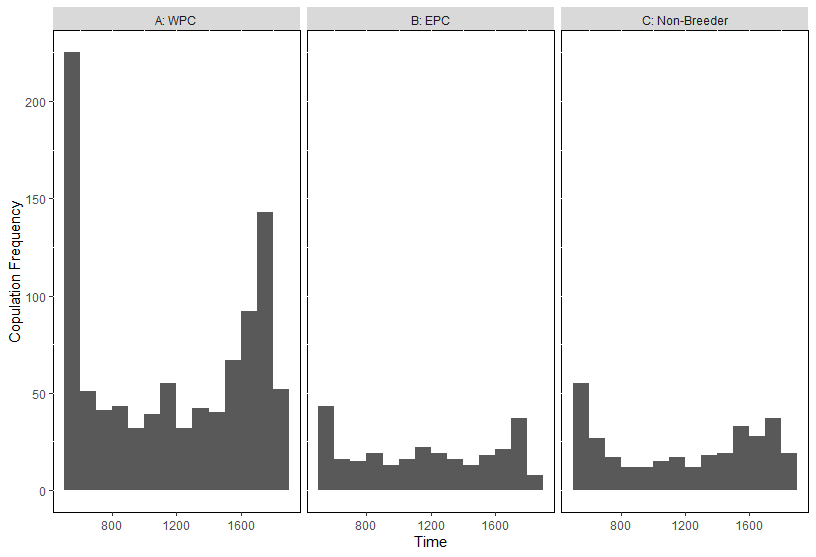

Supplement: S1 Fig — Copulations outside of these hours are rare (see main text). (PNG) [file pone.0324762.s001.png]
