# Supplementary material for: Female sexual agency and frequent extra-pair copulations, but no extra-pair paternity, in Nazca boobies (Sula granti)
Source: PLoS One. 2025 Oct 30;20(10):e0324762. doi: 10.1371/journal.pone.0324762 (PMC12574908; doi:10.1371/journal.pone.0324762)
Supplement: S1 Table — 4-d blocks are named by the first day in the block. By performing the same statistical test for each year, we increased the probability of falsely rejecting a null hypothesis. We used the false discovery rate procedure [48], which controls the expected fraction of null hypotheses that are mistakenly rejected, to appropriately adjust observed P values for this problem. The procedure ranks the n comparisons in order of decreasing P values and compares P values to a critical significance level, beginning with the largest P value. The critical significance level for each comparison, di, is calculated by dividing the specific comparison i by the total number of comparisons n and then multiplying by the false discovery rate (the expected proportion of null hypotheses mistakenly rejected). For example, in Table 1a, the fifth comparison (the comparison with the fifth largest P value) of 11 total comparisons, given a false discovery rate of 0.05, has a di of 0.023 (= 5/11 x 0.05). The achieved significance level (0.08) is > di for that comparison, so the null hypothesis is not rejected for that comparison [48,49]. For the fourth comparison, the achieved significance level of 0.007 is less than the fourth di (0.018), so the null hypothesis is rejected for that comparison and for all subsequent comparisons [48,49]. Rejections of null hypothesis are indicated by bold font. (DOCX) [file pone.0324762.s002.docx]

| **1a. Wilcoxon Tests for 4-day blocks of WPCs and EPCs** | | | | | | | | |
| --- | --- | --- | --- | --- | --- | --- | --- | --- |
| 4-d Block Before Laying | Wilcoxon *P* |  | 4-d Block Before Laying | Wilcoxon *P* |  | 4-d Block Before Laying | Wilcoxon *P* | d_i_ |
| -44 | 0.89 |  | -4 | 3 * 10^-7^ |  | -4 | **3 * 10^-7^** | 0.005 |
| -40 | 1 |  | -8 | 2 * 10^-6^ |  | -8 | **2 * 10^-6^** | 0.009 |
| -36 | 0.5 |  | -20 | 0.002 |  | -20 | **0.002** | 0.014 |
| -32 | 0.15 |  | -16 | 0.007 |  | -16 | **0.007** | 0.018 |
| -28 | 0.46 | sort by *P* | -12 | 0.08 | compare each *P* to | -12 | 0.08 | 0.023 |
| -24 | 0.67 | value | -32 | 0.15 | to critical | -32 | 0.15 | 0.027 |
| -20 | 0.002 |  | -28 | 0.46 | significance | -28 | 0.46 | 0.032 |
| -16 | 0.007 |  | -36 | 0.5 | level d_i_ | -36 | 0.5 | 0.036 |
| -12 | 0.08 |  | -24 | 0.67 |  | -24 | 0.67 | 0.041 |
| -8 | 2 * 10^-6^ |  | -44 | 0.89 |  | -44 | 0.89 | 0.045 |
| -4 | 3 * 10^-7^ |  | -40 | 1 |  | -40 | 1 | 0.050 |
|  | | | | | | | | |
| **1b. Wilcoxon Tests for 4-day blocks of WPCs and EPCs, using only females with complete copulatory histories from blocks -28 through -4** | | | | | | | | |
| 4-d Block Before Laying | Wilcoxon *P* |  | 4-d Block Before Laying | Wilcoxon *P* |  | 4-d Block Before Laying | Wilcoxon *P* | d_i_ |
| -28 | 0.46 |  | -4 | 2 * 10^-4^ |  | -4 | 2 * 10^-4^ | 0.007 |
| -24 | 0.48 |  | -8 | 1 * 10^-4^ | compare each *P* to | -8 | 1 * 10^-4^ | 0.014 |
| -20 | 0.02 | sort by *P* value | -20 | 0.02 | to critical | -20 | 0.02 | 0.021 |
| -16 | 0.07 |  | -16 | 0.07 | significance | -16 | 0.07 | 0.029 |
| -12 | 0.19 |  | -12 | 0.19 | level d_i_ | -12 | 0.19 | 0.036 |
| -8 | 1 * 10^-4^ |  | -28 | 0.46 |  | -28 | 0.46 | 0.043 |
| -4 | 2 * 10^-4^ |  | -24 | 0.48 |  | -24 | 0.48 | 0.050 |
|  | | | | | | | | |
| **1c. Wilcoxon Tests for 4-day blocks of WPCs and EPCs, using only blocks with at least one copulation** | | | | | | | | |
| 4-d Block Before Laying | Wilcoxon *P* |  | 4-d Block Before Laying | Wilcoxon *P* |  | 4-d Block Before Laying | Wilcoxon *P* | d_i_ |
| -44 | 0.89 |  | -4 | 3 * 10^-7^ |  | -4 | **3 * 10^-7^** | 0.005 |
| -40 | 1 |  | -8 | 2 * 10^-6^ |  | -8 | **2 * 10^-6^** | 0.009 |
| -36 | 0.5 |  | -20 | 0.0024 |  | -20 | **0.0024** | 0.014 |
| -32 | 0.15 |  | -16 | 0.0066 |  | -16 | **0.0066** | 0.018 |
| -28 | 0.46 | sort by *P* | -12 | 0.08 | compare each *P* to | -12 | 0.08 | 0.023 |
| -24 | 0.67 | value | -32 | 0.15 | to critical | -32 | 0.15 | 0.027 |
| -20 | 0.002 |  | -28 | 0.46 | significance | -28 | 0.46 | 0.032 |
| -16 | 0.007 |  | -36 | 0.5 | level d_i_ | -36 | 0.5 | 0.036 |
| -12 | 0.08 |  | -24 | 0.67 |  | -24 | 0.67 | 0.041 |
| -8 | 2 * 10^-6^ |  | -44 | 0.89 |  | -44 | 0.89 | 0.045 |
| -4 | 3 * 10^-7^ |  | -40 | 1 |  | -40 | 1 | 0.050 |
|  |  |  |  |  |  |  |  |  |
| **1d. Wilcoxon Tests for 4-day blocks of WPCs and EPCs, using only blocks from females with at least one EPC before starting her clutch** | | | | | | | | |
| 4-d Block Before Laying | Wilcoxon *P* |  | 4-d Block Before Laying | Wilcoxon *P* |  | 4-d Block Before Laying | Wilcoxon *P* | d_i_ |
| -44 | 0.71 |  | -4 | 2 * 10^-4^ |  | -4 | **2 * 10^-4^** | 0.005 |
| -40 | 0.59 |  | -8 | 9 * 10^-4^ |  | -8 | **9 * 10^-4^** | 0.009 |
| -36 | 0.94 |  | -20 | 0.03 |  | -20 | 0.03 | 0.014 |
| -32 | 0.55 |  | -24 | 0.09 |  | -24 | 0.09 | 0.018 |
| -28 | 0.96 | sort by *P* | -16 | 0.09 | compare each *P* to | -16 | 0.09 | 0.023 |
| -24 | 0.09 | value | -12 | 0.5 | to critical | -12 | 0.5 | 0.027 |
| -20 | 0.03 |  | -32 | 0.55 | significance | -32 | 0.55 | 0.032 |
| -16 | 0.09 |  | -40 | 0.59 | level d_i_ | -40 | 0.59 | 0.036 |
| -12 | 0.5 |  | -44 | 0.71 |  | -44 | 0.71 | 0.041 |
| -8 | 9 * 10^-4^ |  | -36 | 0.94 |  | -36 | 0.94 | 0.045 |
| -4 | 2 * 10^-4^ |  | -28 | 0.96 |  | -28 | 0.96 | 0.050 |
