# Supplementary material for: Female sexual agency and frequent extra-pair copulations, but no extra-pair paternity, in Nazca boobies (Sula granti)
Source: PLoS One. 2025 Oct 30;20(10):e0324762. doi: 10.1371/journal.pone.0324762 (PMC12574908; doi:10.1371/journal.pone.0324762)
Supplement: S2 Table — (DOCX) [file pone.0324762.s003.docx]

| Parameter | Estimate | Standard Error | Z value | Pr(>\|z\|) |
| --- | --- | --- | --- | --- |
| Intercept | -1.972 | 1.014 | -1.95 | 0.05 |
| EPCs in the last 16 days | 0.169 | 0.107 | 1.58 | 0.11 |
| WPCs in the last 16 days | 0.112 | 0.072 | 1.57 | 0.12 |
