# Supplementary material for: Female sexual agency and frequent extra-pair copulations, but no extra-pair paternity, in Nazca boobies (Sula granti)
Source: PLoS One. 2025 Oct 30;20(10):e0324762. doi: 10.1371/journal.pone.0324762 (PMC12574908; doi:10.1371/journal.pone.0324762)
Supplement: Attachment — Submitted filename: Response to Reviewers.docx [file pone.0324762.s005.docx]

Response to Reviewers

As you will see, the lone reviewer of your manuscript (no other reviews could be acquired in spite of over a dozen invitations) was very enthusiastic about the importance of this contribution to the literature on extra-pair copulation in birds, particularly considering the complete sexual agency demonstrated by females. In fact, this reviewer recommended acceptance with no further changes.

As Academic Editor, I conducted my own independent review of the manuscript, with my comments appearing below. Please pay careful attention to each of these comments in crafting your revisions.

Academic Editor comments:

Before proceeding to the review comments, we want to thank the editor for taking on the significant extra work of acting as a peer reviewer, in the interest of moving the review process along. We appreciate and acknowledge the extra effort.

Lines 119-120: Did unpaired males simply roam the study plot or did they try to associate themselves with a nest?

Giving more information on the colony social environment would be helpful, yes. To address the comment in the context of both sexes, we have added the following information:

Revision L. 108 “At night, dawn, and dusk the density of boobies in the plot was high, confining birds to individual nest sites in pairs or alone. During daylight hours most birds leave the colony to forage; the remaining males and females have more freedom of movement, although mostly within our plot. Nonetheless, most birds spent most of their time at a nest site while in the plot during the day, occasionally moving to visit other birds or nest sites or to gather nest material. Most males had a single nest site that they occupied and defended during day and night, but males may visit other nest sites when those are undefended during the day, displaying to females and interacting with them there.”

Revision L. 121 “Most of the remaining sites were defended by males that did not acquire a mate that season.”

Revision L. 339: “Birds of each sex were able move around the plot during daylight.”

Lines 179-180: Please clarify what results were correlated, since an r of 0.74 wouldn’t be very reassuring if you’re referring to the assignment of parentage.

We have added new explanation at revision L. 186, clarifying how the correlation was about evaluating *Hin*FI as a proxy for *Hae*III for one problematic gel, the gel for which *Hae*III failed.

Lines 218-219: How were times of day categorized? Also, were multiple days for each female included in the analysis? If so, some sort of comparison among females should first be conducted to rule out non-independence of observations within females.

Revision L 229: To make the time of day categorization clearer we have changed the original text that was at Lines 218–219

“The times of day of WPCs and EPCs were compared using the *chisq-test* R function.”

to

“The times of day of WPCs and EPCs, summed within 1-h bins, were compared using the *chisq-test* R function.”

To address the comment about multiple days, we have added a new supporting analysis using only females that contributed to all 4-d blocks from the -28 d block through the -4 d block (no partial histories, no opportunity for idiosyncratic females contributing only to later blocks to unduly influence that block’s result. The new paragraph describing this result begins on revision L. 392 and involves new S1b Table. The outcome was the same as for the original analysis: WPC frequency exceeds EPC frequency in the 4 d blocks immediately preceding laying.

Line 231: by “the Chi-square test within ANOVA,” do you mean analysis of deviance using the deviance statististic, G2, which is distributed as a Chi-square?

Revision L 242: We have changed the original text that was at Line 237 to

“using the analysis of deviance (the difference in model deviances is distributed as a Chi-square) provided by the package.”

This is consistent with the R documentation that we consulted and describes accurately how the package makes the comparison.

Lines 234-235: This feels like a result more than a method. I would suggest making this statement in the Results.

This refers to the sentence “Finding that the simple model was not significantly different from the complex model (*P* > 0.20), we used the simple model for inference.”

We hear what you’re saying, but consider that this test was not about an inference regarding a hypothesis of scientific interest. Instead, it is just deciding which version of a (small) family of models to use for inference later about scientific hypotheses. This method-relevant result flows nicely in the reader experience in the Methods where it is now, and if instead in the Results, around 170 lines later, we would need to add text to remind the reader what was done and what the outcome means. Also, some readers would be confused into thinking that the outcome has to do with scientific hypotheses of interest, and it does not. We think that the reader’s experience is better in the original, and we have retained that in the revision, pending your reaction.

Lines 275-277: It’s not entirely clear what this Chi-square test was evaluating. Was it a comparison of the number of EPCs vs. WPCs taking place by time period?

We’ve clarified this at the revision’s L. 288, changing

“… (χ2 = 28.1, df = 13, P < 0.01; S1 Fig).” to

“… (comparison of WPC and EPC frequencies by time period, χ2 = 28.1, df = 13, P < 0.01; S1 Fig).”

Lines 327-328: Were there EPCs observed for females that did not occupy a single home nest site around the date of the copulation?

Yes, there were. This analysis at the second paragraph of the subsection “ To What Degree Do Females Control Copulations?” is about the subset of females that can demonstrate obvious intentionality by leaving their habitual consort male to copulate with another at a different location. This subset numbers 132 EPCs. The total number of EPCs is 359, and includes females that spent at least a little time at some other male’s nest site around the time of the EPC, making forays not so obviously an intentional act to get a copulation. The entire set of 359 EPCs are used in later analyses. To address the comment, we have changed the text at revision L. 340 from

“In 132 of the EPCs, the female occupied a single home nest site around the date of the copulation…” to

“In 132 of the 359 EPCs, the female occupied a single home nest site around the date of the copulation…”

Fig. 4: The y-axis label should report the log-transformation.

Done.

Lines 437-440: Is it fair to call it sexual conflict when the EPC behavior of females apparently results in no negative consequence to male fitness?

We’re glad that you made this point. The text could definitely be better on this point. The paragraph that you mentioned makes the point that EPCs must be happening in most birds species, even when they may be seldom seen, because EPFs are observed. Thus, over paternity, a sexual conflict must exist. You could say that no conflict exists in Nazca boobies, but the next sentence (revision L. 465) makes clear that males may have additional reasons to oppose EPCs. We have added to the paragraph that begins on revision L. 464 to make clear that EPCs may impose costs on *Nazca booby males* other than paternity costs. This is not the point of either of these paragraphs about female sexual agency, and instead is a tangential aspect, but it (does sexual conflict exist in Nazca boobies) could have been motivated better, and we hope that it is now. We have also added nuance to revision L. 40 along the same line.

[Note: HTML markup is below. Please do not edit.]

Reviewer's Responses to Questions

We have not responded to any of these reviewer reactions because they did not call for changes.

Comments to the Author

1. Is the manuscript technically sound, and do the data support the conclusions?

The manuscript must describe a technically sound piece of scientific research with data that supports the conclusions. Experiments must have been conducted rigorously, with appropriate controls, replication, and sample sizes. The conclusions must be drawn appropriately based on the data presented.

Reviewer #1: Yes

2. Has the statistical analysis been performed appropriately and rigorously?

Reviewer #1: Yes

3. Have the authors made all data underlying the findings in their manuscript fully available?

Reviewer #1: Yes

4. Is the manuscript presented in an intelligible fashion and written in standard English?

Reviewer #1: Yes

5. Review Comments to the Author

Reviewer #1: • What are the main claims of the paper and how significant are they for the discipline?

This paper is extremly important for behavioural ecology. This is the first robust evidence of complete sexual agency in a female bird aside from lek mating systems.

• Are the claims properly placed in the context of the previous literature? Have the authors treated the literature fairly?

I did not find any irregularities

• Do the data and analyses fully support the claims? If not, what other evidence is required?

The obtained results are so extraordinary that the authors took into account all possible sources of error. Therefore, I have no comments regarding the data and analyses.

• If the paper is considered unsuitable for publication in its present form, does the study itself show sufficient potential that the authors should be encouraged to resubmit a revised version?

I don't know how this article could be improved. The relationship of EPC to future mate choice should be a topic of future work by this team.

6. Do you want your identity to be public for this peer review? For information about this choice, including consent withdrawal, please see our Privacy Policy.

Reviewer #1: Yes: Dariusz Wysocki
